# Xylanase improves the intestinal barrier function of Nile tilapia (*Oreochromis niloticus*) fed with soybean (*Glycine max*) meal

**DOI:** 10.1186/s40104-023-00883-8

**Published:** 2023-07-07

**Authors:** Tong Wang, Nannan Zhou, Junyi He, Zhenzhen Hao, Chentao Zhou, Yidi Du, Zhenyu Du, Xiaoyun Su, Meiling Zhang

**Affiliations:** 1 Laboratory of Aquaculture Nutrition and Environmental Health (LANEH), School of Life Sciences, East China Normal University, Shanghai, 200241 China; 2 State Key Laboratory of Animal Nutrition, Institute of Animal Sciences, Chinese Academy of Agricultural Sciences, Beijing, 100193 China

**Keywords:** Butyrate, Exogenous enzymes, Intestinal health, Intestinal microbiota, Mucin, Xylanase

## Abstract

**Background:**

Soybean (*Glycine max*) meal is one of the important protein sources for fish, but the non-starch polysaccharides (NSP) in soybean meal impair the intestinal barrier function. Here we aimed to investigate whether xylanase can alleviate the adverse effects on the gut barrier induced by soybean meal in Nile tilapia and to explore the possible mechanism.

**Results:**

Nile tilapia (*Oreochromis niloticus*) (4.09 ± 0.02 g) were fed with two diets including SM (soybean meal) and SMC (soybean meal + 3,000 U/kg xylanase) for 8 weeks. We characterized the effects of xylanase on the gut barrier, and the transcriptome analysis was performed to investigate the underlying mechanism. Dietary xylanase improved intestinal morphology and decreased the concentration of lipopolysaccharide (LPS) in serum. The results of transcriptome and Western blotting showed that dietary xylanase up-regulated the expression level of mucin2 (MUC2) which may be related to the inhibition of protein kinase RNA-like endoplasmic reticulum kinase (*perk*)/activating transcription factor 4 (*atf4*) signaling pathways. Microbiome analysis showed that addition of xylanase in soybean meal altered the intestinal microbiota composition and increased the concentration of butyric acid in the gut. Notably, dietary sodium butyrate was supplemented into the soybean meal diet to feed Nile tilapia, and the data verified that sodium butyrate mirrored the beneficial effects of xylanase.

**Conclusions:**

Collectively, supplementation of xylanase in soybean meal altered the intestinal microbiota composition and increased the content of butyric acid which can repress the *perk*/*atf4* signaling pathway and increase the expression of *muc2* to enhance the gut barrier function of Nile tilapia. The present study reveals the mechanism by which xylanase improves the intestinal barrier, and it also provides a theoretical basis for the application of xylanase in aquaculture.

## Background

Soybean (*Glycine max*) meal is commonly used in aquaculture due to its high protein level, low cost, relatively well balanced-amino acid profile and widespread availability [1, 2]. In view of the above advantages, soybean meal is an excellent protein source for aquatic feeds [3]. However, soybean meal also has adverse effects on fish gut health, especially on the structure and barrier function [4–6]. Various antinutritional factors (ANFs) contained in soybean meal have been identified, including saponins, lectins, antigenic protein, phytic acid and trypsin inhibitor, and many studies have focused on the negative effects of glycinin and β-conglycinin on gut health in aquatic animals [7–10]. In fact, non-starch polysaccharides (NSP) in soybean meal also act as the ANFs to cause intestinal barrier dysfunction [11, 12], but little attention has been paid to the negative effects of NSP on fish.

NSP, the leguminous cell wall structure, are main constituents of cereals [13]. NSP in diet can increase the digesta viscosity and retard the intestinal transit time, which in turn influenced nutrient availability and the microbial composition in the intestine [12, 14]. Previous studies indicated that dietary NSP reduced the villus height of the small intestine and inhibited the growth of beneficial bacteria [15, 16]. In addition, the animals fed with a diet containing high levels of NSP showed programmed death of epithelial cells [17]. Eliminating the adverse effects of NSP on host health is essential for the application of plant protein in aquatic feeds.

Plant ingredients in aquatic feeds contained a certain amount of NSP, and it was reported that soybean meal contains 217 g/kg NSP [11]. In aquaculture, soybean meal or 5 g/kg NSP in the diet reduced the availability of nutrients, impaired the integrity of the distal intestine and decreased the length of intestinal villus [18–20]. Addition of exogenous enzymes is an effective method to attenuate the adverse effects of NSP on host health. Xylanase is one of the NSP degradation enzymes and can specifically hydrolyze xylan [21]. However, xylanase is scarce or absent in the intestine of fish [22]. In aquatic animals, the addition of xylanase in the diet decreased the viscosity of digesta, improved the growth performance, increased the nutrient digestibility and protein efficiency ratio [23–25]. 0.5 g/kg dietary xylanase in soybean meal improved the growth performance, feed utilization, and digestive enzyme activity in Nile Tilapia [26]. Supplementation of xylanase in sunflower meal alleviated NSP-induced lower apparent digestibility [27]. Previous study has reported that dietary xylanase administration improved the structure of intestinal microvillus and subsequently increased the growth performance of large yellow croaker (*Larimichthys crocea*) [28]. Multiple studies have indicated the beneficial effects of xylanase, but the underlying mechanism remains unclear.

Xylanase can hydrolyze xylan in diet and produce oligomers including xylooligosaccharides (XOS) with varying degrees of polymerization. The released oligomers can be fermented by gut microbiota to support the growth of certain beneficial gut microbes by exerting prebiotic-like effects [29, 30]. It has been reported that dietary xylanase reduced the amount of *Aeromonas* and *Escherichia coli* and increased the abundance of *Lactobacillus* in the gut of Jian carp (*Cyprinus carpio* var. Jian) [31]. Furthermore, the addition of dietary xylanase in wheat–soybean meal-based diets increased the fecal *Lactobacillus* populations and inhibited the fecal *Escherichia coli* populations, which thereby improved the growth performance in growing pigs [32]. All these researches suggested that xylanase can modulate the gut microbiota composition. Gut microbiota can ferment xylanase-released oligomers to produce metabolites such as short-chain fatty acids (SCFAs) which have beneficial effects on host health. Supplementation of carbohydrase enzymes containing xylanase in a wheat–soybean meal-based diet enhanced the levels of acetate and total SCFAs in the ileal and caecal digesta of broiler [33]. Dietary xylanase addition in plant protein-enriched diets altered the intestinal microbiota composition and increased the concentration of acetate, propionate and butyrate in grass carp (*Ctenopharyngodon idella*) [34]. SCFAs, especially butyrate, can act as energy source to maintain the mucosal barrier in the gut [35, 36]. However, the potential link of the beneficial effects of xylanase, the intestinal microbiota and SCFAs remains unclear.

Nile tilapia (*Oreochromis niloticus*) is one of the most important economically omnivorous fish species and is an ideal fish model for understanding the mechanism of a specific diet additive due to its rapid growth and available genomic information [37]. Soybean meal causes negative effects on intestinal health in fish [38]. To investigate whether and how xylanase alleviates the negative effects induced by soybean meal on the gut barrier, Nile tilapia were fed with the soybean meal diet supplemented with or without dietary xylanase, and gut morphology, transcriptome analysis, and gut microbiota composition were detected.

## Methods

### Experimental animals and diets

In the first experiment, 300 Nile tilapia were collected from Tianfa Fishery Development Co., Ltd. (Guangzhou, China). Nile tilapia were fed with a commercial feed (Tongwei Co., Ltd., Chengdu, China) in 400-L tanks for two weeks. Then 180 healthy Nile tilapia with no exterior injuries and deformities (initial weight: 4.09 ± 0.02 g) were randomly divided into six tanks (30 fish per tank) in the indoor-recirculating system (200-L tanks), fed with two diets including soybean meal diet (SM) or soybean meal diet supplemented with 3,000 U/kg xylanase (SMC). The fish were hand-fed twice a day at 09:00 and 18:00 for 8 weeks. The feeding rate was 4% of body weight and the feed consumption of fish was adjusted according to the batch weighing every two weeks.

In the second experiment, two hundred Nile tilapia were purchased from Tianfa Fishery Development Co., Ltd. (Guangzhou, China), and fish were acclimated in four 400-L tanks. After 2 weeks, 120 healthy Nile tilapia were randomly distributed into two groups (60 fish in each treatment, each treatment contains three tanks) and fed with the soybean meal diet (SM) or soybean meal diet supplemented with 40 mmol/kg sodium butyrate (SB) for 6 weeks. Nile tilapia were cultured in 200-L tanks and fed two times per day according to 4% of body weight. The fish were cultured on a natural photoperiodicity (12 h light–12 h dark) and the water temperature was 27 ± 1 ºC. During the feeding trial, the pH ranged from 7.5 to 7.9. Ammonia nitrogen and dissolved oxygen were 7.8 to 8.2 and 0.05 to 0.08 mg/L, respectively.

The iso-nitrogenous and iso-lipidic diets were formulated. Protein sources were casein, fish meal, and soybean meal. Corn starch was used as the carbohydrate source. Lipid sources were fish oil and soybean oil. For the diet preparation, individual ingredient was mixed with oil and distilled water, then extruded into 2 μm pellets (Tables 1 and 2). The feeds were dried in the air and then stored at −20 °C until use. The dried feed was used to detect the content of crude protein and crude lipid. Crude protein was determined using a semi-automatic Kjeldahl System (FOSS, Sweden) following the previous study [39]. Crude lipid was analyzed following the methods of chloroform–methanol (2:1, v:v) extraction [40].

**Table 1:** The diet formulation for the first experiment

Ingredients, g/kg	SM	SMC
Fish meal (670 g/kg protein)	75	75
Casein (900 g/kg protein)	120	120
Soybean meal (460 g/kg protein)	400	400
Corn starch	300	300
Fish oil	13.25	13.25
Soybean oil	27	27
Vitamin premix^1^	12	12
Mineral premix^2^	12	12
Carboxymethyl cellulose (CMC)	10	10
Cellulose	8.5	8.5
Butylated hydroxytoluene	0.25	0.25
Choline chloride	5	5
Ca(H_2_PO_4_)_2_	15	15
Dimethyl-beta-propiothetin	2	2
Xylanase	0	3,000U
Total quantity	1,000	1,000
Crude lipid	4.93	4.96
Crude protein	33.8	33.4

^1^Mixed vitamin (g/kg): Inositol 25 g, Cholin 100 g, Vitamin B_12_ 5 g, Niacin 25 g, Vitamin B_6_ 5 g, Folic acid 1 g, Vitamin B_2_ 5 g, Biotin 0.25 g, Vitamin B_1_ 5 g, Vitamin C 10 g, Vitamin K_3_ 1 g, Vitamin A 0.15 g, Vitamin E 2.5 g, Pantothenic acid 10 g, Vitamin D_3_ 0.00125 g

^2^Mixed minerals (g/kg): NaSeO_3_ 0.02 g, MgSO_4_·7H_2_O 147.4 g, NH_4_ molybdate 0.06 g, CoCl_2_·6H_2_O 0.08 g, NaCl 49.8 g, KI 0.16 g, Fe (II) gluconate 10.9 g, CuSO_4_·5H_2_O 0.62 g, ZnSO_4_·7H_2_O 4.67 g, MnSO_4_·H_2_O 3.12 g

**Table 2:** The diet formulation for the second experiment

Ingredients, g/kg	SM	SB
Fish meal (670 g/kg protein)	75	75
Casein (900 g/kg protein)	120	120
Soybean meal (460 g/kg protein)	400	400
Corn starch	300	300
Fish oil	13.25	13.25
Soybean oil	27	27
Vitamin premix^1^	12	12
Mineral premix^2^	12	12
Carboxymethyl cellulose (CMC)	10	10
Cellulose	8.5	8.5
Butylated hydroxytoluene	0.25	0.25
Choline chloride	5	5
Ca(H_2_PO_4_)_2_	15	15
Dimethyl-beta-propiothetin	2	2
Sodium butyrate, mmol/kg	0	40
Total quantity	1,000	1,000
Crude lipid	4.93	4.96
Crude protein	33.8	33.4

^1^Mixed vitamin (g/kg): Inositol 25 g, Cholin 100 g, Vitamin B_12_ 5 g, Niacin 25 g, Vitamin B_6_ 5 g, Folic acid 1 g, Vitamin B_2_ 5 g, Biotin 0.25 g, Vitamin B_1_ 5 g, Vitamin C 10 g, Vitamin K_3_ 1 g, Vitamin A 0.15 g, Vitamin E 2.5 g, Pantothenic acid 10 g, Vitamin D_3_ 0.00125 g

^2^Mixed minerals (g/kg): NaSeO_3_ 0.02 g, MgSO_4_·7H_2_O 147.4 g, NH_4_ molybdate 0.06 g, CoCl_2_·6H_2_O 0.08 g, NaCl 49.8 g, KI 0.16 g, Fe (II) gluconate 10.9 g, CuSO_4_·5H_2_O 0.62 g, ZnSO_4_·7H_2_O 4.67 g, MnSO_4_·H_2_O 3.12 g

### Sample collection and proximate analysis of whole fish

At the end of the experiment, Nile tilapia were weighed after fasting for 12 h. Twelve fish from each group were randomly selected and anesthetized with 20 mg/L MS-222 (tricaine methanesulfonate, Sigma-Aldrich, Darmstadt, Germany). Blood was drawn from the caudal vein with heparinized 1-mL syringes (Taizhou Kl Medical Equipment Co., Ltd., Taizhou, China). Then the blood was immediately centrifuged at 3,000 r/min, 4 ºC for 10 min, and the separated serum was stored at −80 ºC for enzyme-linked immunosorbent assay (ELISA) analysis. The whole intestine was collected and stored at −80 ºC. The gut content was collected for gut microbial composition and short-chain fatty acids (SCFAs) analysis. The weight of trunk, viscera, intraperitoneal fat and liver of each fish was recorded and the organ indices were calculated following the under formula:

$$\mathrm{Weight}\;\mathrm{gain}\;(\mathrm g)=\mathrm{Final}\;\mathrm{fish}\;\mathrm{weight}\;(\mathrm g)-\mathrm{Initial}\;\mathrm{fish}\;\mathrm{weight}\;(\mathrm g)$$

$$\mathrm{Feed}\;\mathrm{conversion}\;\mathrm{ratio}=\mathrm{Feed}\;\mathrm{given}\;(\mathrm g)\;/\;\mathrm{Weight}\;\mathrm{gain}\;(\mathrm g)$$

$$\mathrm{Carcass}\;\mathrm{ratio}\;(\%)=100\;\times\;\lbrack\mathrm{Total}\;\mathrm{body}\;\mathrm{weight}\;(\mathrm g)-\mathrm{head}\;\mathrm{weight}\;(\mathrm g)-\mathrm{fin}\;\mathrm{weight}\;(\mathrm g)-\mathrm{visceral}\;\mathrm{weight}\;(\mathrm g)\rbrack\;/\;\mathrm{Final}\;\mathrm{fish}\;\mathrm{weight}\;(\mathrm g)$$

$$\mathrm{Viscera}\;\mathrm{index}\;(\%)=100\times\left[\mathrm{Viscera}\;\mathrm{weight}\;(\mathrm g)\;/\;\mathrm{body}\;\mathrm{weight}\;(\mathrm g)\right]$$

$$\mathrm{Hepatosomatic}\;\mathrm{index}\;(\%)=100\times\left[\mathrm{Liver}\;\mathrm{weight}\;(\mathrm g)\;/\;\mathrm{body}\;\mathrm{weight}\;(\mathrm g)\right]$$

$$\mathrm{Mesenteric}\;\mathrm{fat}\;\mathrm{index}\;(\%)=100\;\times\;\left[\mathrm{Mesenteric}\;\mathrm{fat}\;\mathrm{weight}\;(\mathrm g)\;/\;\mathrm{body}\;\mathrm{weight}\;(\mathrm g)\right]$$

### Enzyme-linked immunosorbent assay (ELISA)

The content of LPS in serum was measured by a fish lipopolysaccharide (LPS) kit according to the relevant protocol (HB794-QT, Shanghai Hengyuan Biotechnology Co., Ltd., Shanghai, China).

### Histochemical staining and immunohistochemistry

The intestine was sampled and frozen with liquid nitrogen and then fixed in 4% formaldehyde. The intestine was sliced transversely into 5 μm sections and then stained with hematoxylin and eosin (H&E). The stained samples were photographed by using a Nikon Microscope Eclipse Ni (Nikon Corporation, Tokyo, Japan). Villus width and villus length were measured from at least 24 segments in per treatment with an imaging software (Nis-Elements F package version 4.60).

The slides of intestinal tissue were used for immunohistochemical staining of mucin2 (MUC2). Antigen was unmasked in sodium citrate (pH = 6). Slides were blocked with 10% normal rabbit serum and incubated with MUC2 antibody (CY6761, Abways, Shanghai, China) overnight at 4 °C. All slides were incubated with diaminobenzidine solution (K5007, DAKO GmbH, Thüringen, Germany) and horseradish peroxidase-conjugated secondary antibodies (GB23303, Wuhan Servicebio Technology Co., Ltd., Wuhan, China). Hematoxylin staining was performed to visualize nuclei. The stained slides were analyzed by using Nikon Eclipse TS100 (Nikon Corporation, Tokyo, Japan) with a light microscope.

### Quantitative real-time polymerase chain reaction (qRT-PCR)

Total RNA was extracted from the intestine of six fish by using a Trizol (R701-01/02, Nanjing Vazyme Biotech Co., Ltd., Nanjing, China) according to the manufacturer's instructions (*n* = 6). The integrity and purity of RNA were detected using 1% agarose gel electrophoresis and Nanodrop 2000 (Thermo Fisher Scientific, Massachusetts, USA). The A_260_/A_280_ value of RNA ranged from 1.9 to 2.1. cDNA was reversed transcribed from RNA by using the PrimeScript™ RT reagent kit (Nanjing Vazyme Biotech Co., Ltd., Nanjing, China) according to the manufacturer's protocol. Primers were designed in NCBI (Listed in Table 3) for qRT-PCR. Elongation factor 1 alpha (*ef1α*) and *β-actin* were used as the reference genes. The qRT-PCR reaction solution contained 1 μL of cDNA template, 5 μL of 2 × ultra SYBR mixture, 3 μL of ddH_2_O, and 0.25 μL of 10 mmol/L forward and reverse primers. The reaction conditions were 95 °C for 30 s, followed by 40 cycles at 95 °C for 5 s and annealing at 60 °C for 20 s. The qRT-PCR primers efficiency was between 90% and 110%, and the correlation coefficient of each gene was over 0.98. The relative gene expression level was calculated using the comparative (2^−ΔΔCt^) method [41].

**Table 3:** Primer sequences used in qRT- PCR analyses

Gene	Primers	Sequence (5’→3’)	Gene Bank NO
*ef1α*	F	ATCAAGAAGATCGGCTACAACCCT	KJ123689.1
	R	ATCCCTTGAACCAGCTCATCTTGT	
*β-actin*	F	AGCCTTCCTTCCTTGGTATGGAAT	KJ126772.1
	R	TGTTGGCGTACAGGTCCTTACG	
*muc2*	F	ACTACAACTCCCCCTCCTCC	XM_025902524.1
	R	CCCACCCTTCACATACACAGTC	
*nfkb*	F	CGACCACTACCTACACGCTC	XM_019363515.2
	R	GATGTCGTTTGAGGCATCGC	
*il1β*	F	GAGCACAGAATTCCAGGATGAAAG	XM_019365842.2
	R	TGAACTGAGGTGGTCCAGCTGT	
*perk*	F	GATGTTTCAGGGGCAGCTCT	XM_003447769.5
	R	CGTCGTGGGAGAACTTGTCA	
*eif2a*	F	AGAGCTTCTTCAAGGCCGAC	XM_005462748.4
	R	TTGGGCCGTTCTTCGATAGC	
*atf4*	F	CATTGTTCCTCGTTCCTGGG	XM_005464867.4
	R	CAAAAGCATCATCTGCTTTGCCA	
*casp3*	F	GGAGTGGACGATACAGACGCAAA	NM_001282894.1
	R	TGAAGCTGTGTGACTGGGGCTT	

*ef1a* Elongation factor 1 alpha, *muc2* Mucin2, *nfkb* Nuclear factor kappa B, *il1β* Interleukin 1 beta, *perk* Protein kinase RNA-like endoplasmic reticulum kinase, *eif2α* Eukaryotic initiation factor 2 alpha, *atf4* Activating transcription factor 4, *casp3* Caspase3

### Transcriptome analysis of intestine

Total RNA was extracted from the intestine and 1 μg total RNA was used for sequencing by TruSeq mRNA sample prep kit (RS-122–2301, Illumina, Inc., San Diego, USA). Poly-A selected RNA extraction, RNA fragmentation, random hexamer-primed reverse transcription, and paired-end sequencing were subsequently performed on a Hiseq platform (Illumina, Inc., San Diego, USA). The raw reads were pre-processed to filter low-quality reads or adapter sequences, and aligned with the *Oreochromis niloticus* reference genome (GCF_001858045.2) using HISAT v2.1.0. In total, 80,581 transcripts and 38,885 genes were obtained. Aligned reads were assembled into transcripts using StringTie v2.1.2 to estimate their abundance. Differential expression analysis between SM and SMC group was analyzed using DEGSeq2 v1.24.0. Analysis of differentially expressed genes (DEGs) was conducted using Database for Annotation, Visualization and Integrated Discovery (DAVID) v6.8. To systematically analyze the functions of DEGs, Kyoto Encyclopedia of Genes and Genomes (KEGG) pathway analysis was carried out by Goatools (https://github.com/tanghaibao/goatools.). Pathways with adjusted *P* < 0.05 were significantly enriched.

### Western blot

The intestine was homogenized and lysed in ice-cold radioimmunoprecipitation assay buffer containing phenylmethylsulfonyl fluoride (P0013B, Beyotime Biotechnology, Shanghai, China) for 40 min. The protein concentration of the intestine was measured using bicinchoninic acid (BCA) protein assay kit (P0012, Beyotime Biotechnology, Shanghai, China). Total protein was mixed with 5 × sodium dodecyl sulfate loading buffer and boiled at 100 °C for 5 min. 50 μg protein was loaded on sodium dodecyl sulfate polyacrylamide gel electrophoresis and blotted onto nitrocellulose filter membranes (NC; Millipore, Darmstadt, Germany). The NC membranes were blocked by 5% bovine serum albumin and then incubated at 4 °C overnight with the 1:600 diluted antibodies: anti-GAPDH antibody (AF0911, Affinity Biosciences, Ohio, USA), anti-cleaved IL-1 beta antibody (AF4006, Affinity Biosciences, Ohio, USA), anti-MUC2 (CY6761, Abways, Shanghai, China), anti-CHOP (15204–1-AP, Proteintech, Rosemont, USA), anti-Phospho-NF-κB p65 (Ser536) (AF2006, Affinity Biosciences, Ohio, USA), anti-GRP78 (11587–1-AP, Proteintech, Rosemont, USA). The membranes were washed three times with Tris Buffered Saline + Tween 20 (TBST) buffer at room temperature and then incubated with goat anti-rabbit IgG (Li-Cor Biotechnology, Nebraska, USA) or anti-mouse IgG (Li-Cor Biotechnology, Nebraska, USA). The NC membranes were scanned with the Odyssey CLx Imager (Li-Cor Biotechnology, Nebraska, USA).

### Intestinal microbiota analysis

Intestinal content was collected for gut microbiota composition analysis. The genomic DNA of intestinal bacteria was extracted, and the V3–V4 region of 16S rRNA gene was amplified with primers 338F (5'-ACTCCTACGGGAGGCAGCA-3') and 806R (5'-GGACTACHVGGGTWTCTAAT-3'). The amplifications were sequenced on an Illumina MiSeq platform, generating paired-end reads (Shanghai Personal Biotechnology Co., Ltd., Shanghai, China). The paired-end reads were analyzed with QIIME2 for quality filtering, denoising, and chimaera checking. All sequences were clustered to generate non-singleton amplicon sequence variants (ASV) at the 97% similarity level. Alpha diversity metrics including Shannon, Chao1, Simpson, and Goods were calculated by QIIME. Principal component analysis (PCA) was analyzed by R software. The sequences were available in Sequence Read Archive (SRA) (BioProject accession number PRJNA891636).

### Short chain fatty acids analysis

The collected gut content was resuspended with 200 μL distilled water and homogenized for 60 s. Then the samples were acidified with 50 μL 50% sulfuric acid and vortexed for 30 s. The mixture was supplemented with 300 μL diethyl ether to extract SCFAs. The concentration of SCFAs was analyzed by a gas chromatography (Nexis GC-2010 Plus; Shimadzu, Kyoto, Japan) under the conditions as reported by Wang et al. [42]. The concentration of SCFAs was calculated by an external standard method.

### Statistical analysis

All data in the experiment was presented as the mean ± standard error of the mean (SEM). The difference between groups was detected by an Unpaired Student’s *t*-test using GraphPad Prism 9 software. The significant difference between the two groups was presented at *P* < 0.05 (*), or *P* < 0.01 (**).

## Results

### The effects of xylanase on the growth performance

At the end of the feeding trial, the growth indices were recorded to analyze the effects of xylanase on the growth performance of Nile tilapia. The results showed that there was no significant difference in the feed conversion ratio between SM and SMC groups (Table 4), but xylanase supplementation caused a lower weight gain compared with the soybean meal diet (*P* < 0.05, Table 4). In order to identify the main reason for the lower weight gain in SMC group, the body condition indices were detected. Compared with SM group, the addition of dietary xylanase decreased the viscera index significantly, but increased the hepatosomatic index of Nile tilapia (*P* < 0.05, Table 4). A similar level of mesenteric fat index was observed in both groups (Table 4). The carcass ratio was higher in SMC treatment than that in SM treatment, suggesting that addition of xylanase increased the percentage of the edible portion of fish (*P* < 0.05, Table 4). These data showed that xylanase supplementation in soybean meal diet decreased the weight gain of Nile tilapia, but it increased the percentage of the edible portion of fish.

**Table 4:** The effects of xylanase on the growth performance of Nile tilapia

	**SM**	**SMC**
Initial weight, g	4.09 ± 0.02	4.09 ± 0.02
Final weight, g	26.74 ± 0.29^a^	23.85 ± 1.18^b^
Weight gain, g	22.66 ± 0.26^a^	19.77 ± 1.19^b^
Feed conversion ratio	0.86 ± 0.02	0.88 ± 0.04
Viscera index, %	12.92 ± 1.69^a^	10.7 ± 1.29^b^
Hepatosomatic index, %	1.18 ± 0.39^b^	1.72 ± 0.26^a^
Mesenteric fat index, %	0.13 ± 0.15	0.1 ± 0.08
Carcass ratio, %	48.05 ± 2.58^b^	50.67 ± 1.68^a^

*SM *Fish fed with soybean diet, *SMC *Fish fed with soybean diet supplemented with 3,000 U/kg xylanase. Data were expressed as mean ± SEM (*n* = 6)

^a,b^Mean values within a row with unlike superscript letters were significantly different (*P* < 0.05) based on *t*-test

### Dietary xylanase improved the function of the gut barrier

**Fig. 1:**
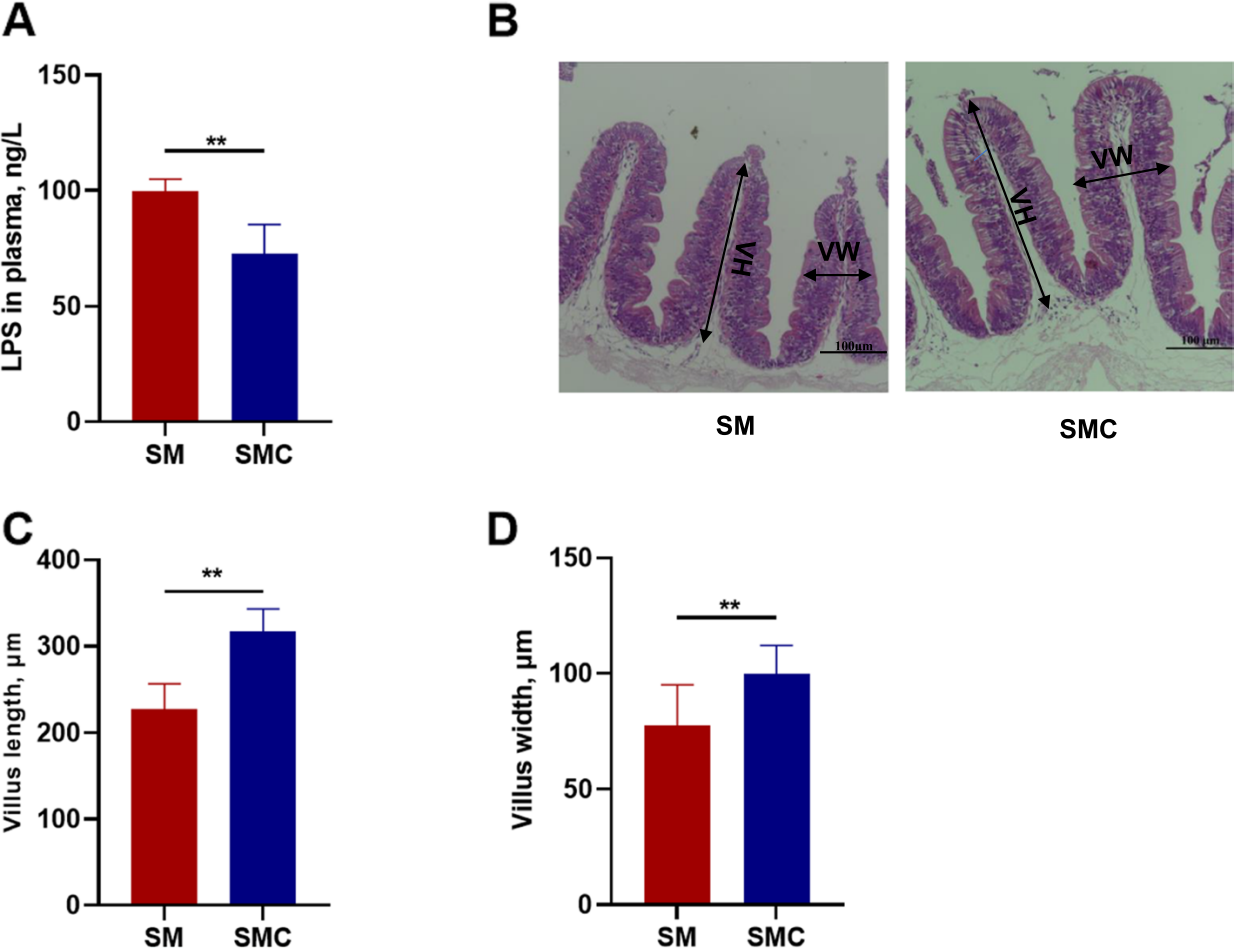
Effects of xylanase on the intestinal integrity and morphology in Nile tilapia. **A** Lipopolysaccharide (LPS) in serum, **B** Hematoxylin and eosin (H&E) staining of intestine, **C** Villus length (VH), **D** Villus width (VW). Data was expressed as mean ± SEM (*n* = 6). SM, fish fed with soybean diet; SMC, fish fed with soybean diet supplemented with 3,000 U/kg xylanase. The significant differences between two groups were presented at *P* < 0.01 (**)

In order to detect whether xylanase improves the gut health of Nile tilapia, we detected the concentration of LPS in serum. The results showed that dietary xylanase significantly decreased LPS content in plasma compared with the SM group (*P* < 0.05, Fig. 1A). H&E staining indicated the villus length and villus width were obviously increased in SMC group compared with SM group (*P* < 0.05, Fig. 1B–D). All these data suggested that the addition of xylanase in soybean meal improved the intestinal structure and integrity.

### Xylanase increased the expression of MUC2 in the gut

**Fig. 2:**
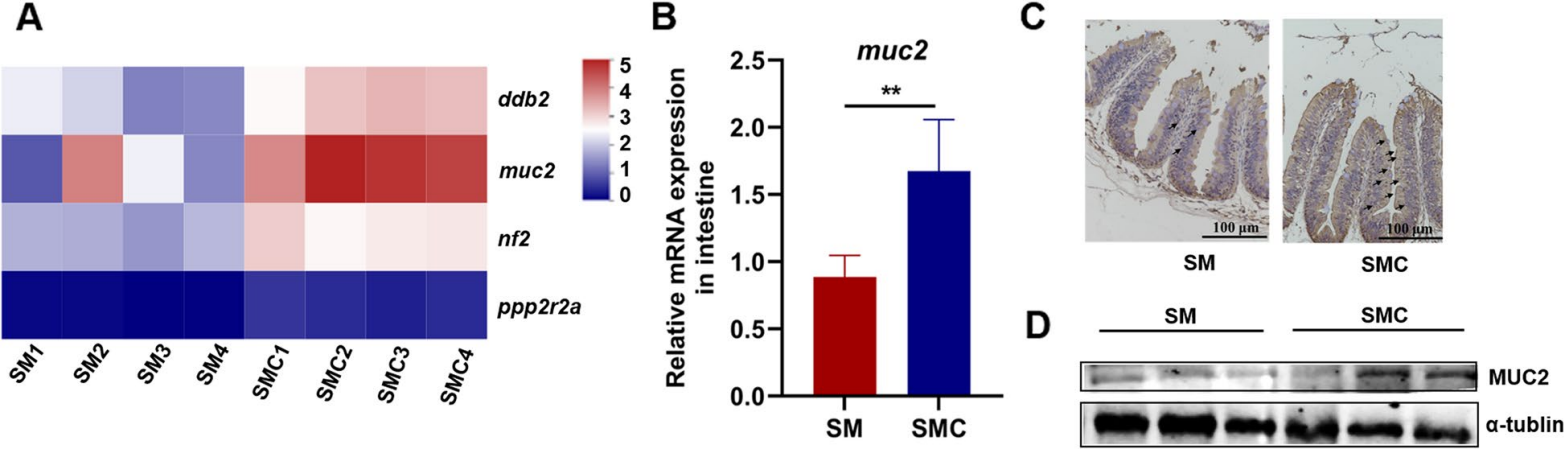
Effects of dietary xylanase on the expression of MUC2 in the gut of Nile tilapia. **A** Heatmap of differentially expressed genes of gut barrier in the comparison between SM vs. SMC (*n* = 4), **B** Gene expression level of mucin2 (*muc2*) (*n* = 6), **C** Immunohistochemical of MUC2 in gut of Nile tilapia (*n* = 3), **D** Western blot analysis of intestinal MUC2 (*n* = 3). Data was expressed as mean ± SEM. SM, fish fed with soybean diet; SMC, fish fed with soybean diet supplemented with 3,000 U/kg xylanase. The significant differences between two group were presented at *P* < 0.01 (**)

To identify the mechanism by which xylanase improved the gut barrier function, the transcriptome analysis was conducted. We screened all gut barrier-related genes and found that the relative expression of mucin 2 (*muc2*) was obviously upregulated by xylanase supplementation (Fig. 2A). The expression level of *muc2* was detected to verify the RNAseq results, and consistently, the expression level of *muc2* was significantly increased in SMC compared with SM (*P* < 0.05, Fig. 2B). The protein level of MUC2 was also detected in the intestine by Western blot and immunohistochemistry analysis. The results confirmed that xylanase administration increased the production of MUC2 in the intestine of Nile tilapia (Fig. 2C and D).

### Supplementation of xylanase relieved the endoplasmic reticulum stress and inflammatory response induced by soybean meal

**Fig. 3:**
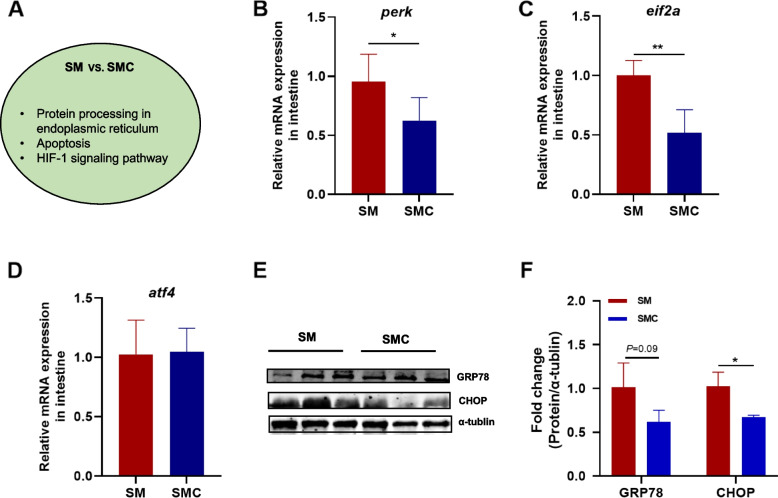
Effects of dietary supplementation with xylanase on endoplasmic reticulum (ER) stress in Nile tilapia. **A** Pathways changed in comparison SM vs. SMC (*n* = 4). Gene level of **B** Protein kinase RNA-like endoplasmic reticulum kinase (*perk*), **C** Initiation Factor 2 alpha (*eif2α*), **D** Activating transcription factor 4 (*atf4*) (*n* = 6), **E** Western blot analysis of intestinal glucose regulatory protein 78 (GRP78) and C/EBP homologous protein (CHOP) (*n* = 3), **F** Quantitation of the levels of GRP78 and CHOP normalised to that of α-tublin (*n* = 3). Data was expressed as mean ± SEM. SM, fish fed with soybean diet; SMC, fish fed with soybean diet supplemented with 3,000 U/kg xylanase. The significant differences between two group were presented at *P* < 0.05 (*), *P* < 0.01 (**)

To explore how xylanase upregulates the expression of MUC2, we used KEGG database to enrich pathways which were significantly changed in xylanase administration. The data showed that the pathway of protein processing in endoplasmic reticulum changed significantly in SMC group compared with SM group (Fig. 3A). Therefore, qPCR analysis was used to identify the expression level of genes related to protein processing in endoplasmic reticulum. The result indicated that the expression level of protein kinase RNA-like endoplasmic reticulum kinase (*perk*) was down-regulated in SMC group (Fig. 3B). The activation of *perk* can trigger unfolded protein response (UPR) pathways including glucose regulatory protein 78 (GRP78), eukaryotic initiation factor 2 alpha (eIF2α), transcription factor 4 (ATF4) and C/EBP homologous protein (CHOP) [43]. In this work, soybean meal increased the expression level of *eif2α*, while xylanase administration down-regulated the gene expression of *eif2α* (*P* < 0.05, Fig. 3C). Neither soybean meal nor xylanase influenced the expression level of *atf4* (Fig. 3D). The addition of xylanase inhibited the protein expression of GRP78 with a decreased tendency (Fig. 3E and F**)**. Compared with SM group, the protein expression of CHOP was significantly downregulated in SMC group (Fig. 3E and F**)**. Collectively, dietary xylanase alleviated endoplasmic reticulum stress induced by soybean meal.

**Fig. 4:**
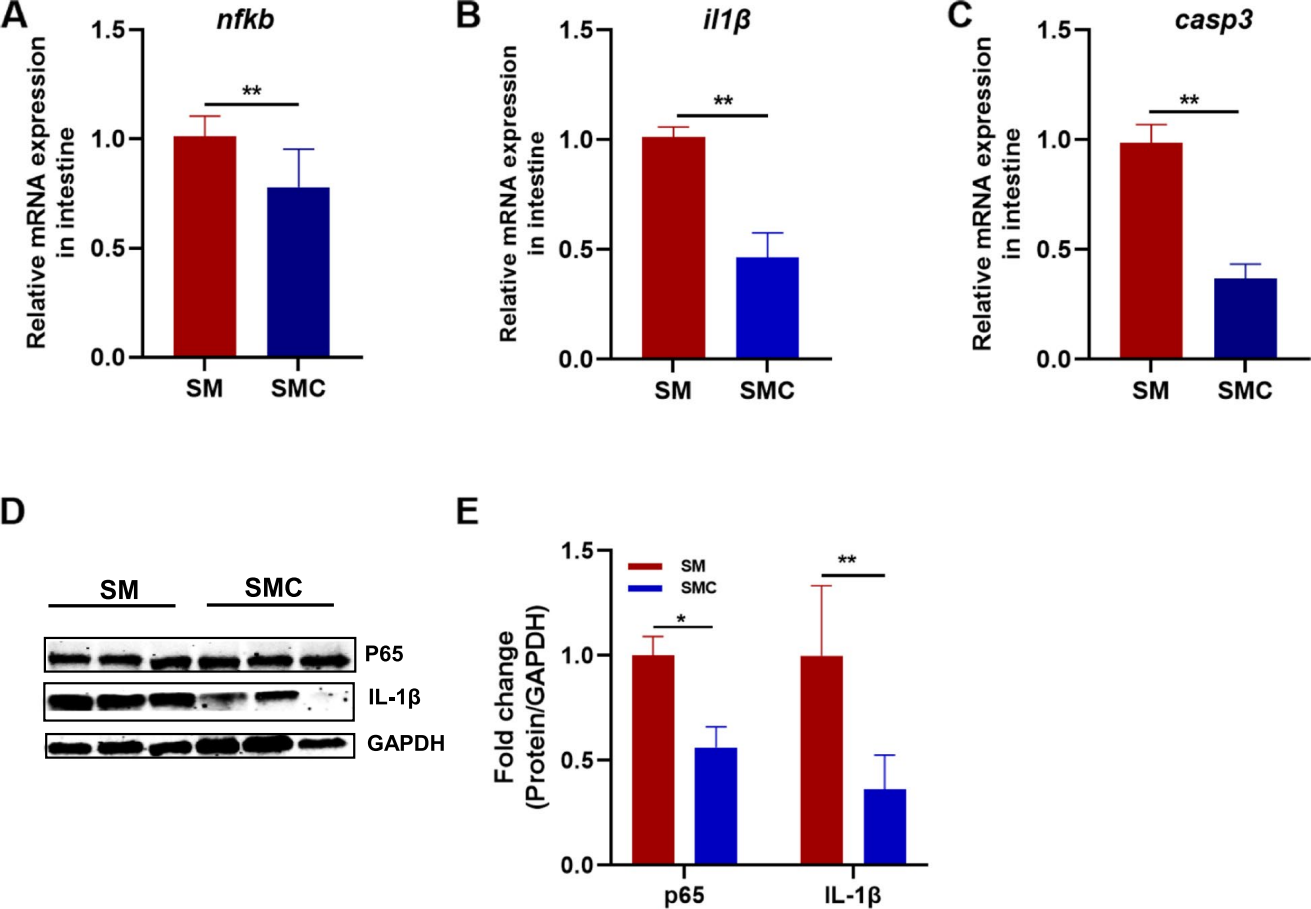
Effects of dietary xylanase on the inflammation response in Nile tilapia. Gene level of **A** Nuclear factor kappa B (*nfkb*), **B** Interleukin 1 beta (*il1β*) and **C** Caspase3 (*casp3*) (*n* = 6), **D** Western blot analysis of intestinal P65 and IL1β (*n* = 3), **E** Quantitation of the levels of P65 and IL1β normalized to that of GAPDH (*n* = 3). Data was expressed as mean ± SEM. SM, fish fed with soybean diet; SMC, fish fed with soybean diet supplemented with 3,000 U/kg xylanase. The significant differences between two group were presented at *P* < 0.05 (*), *P* < 0.01 (**)

According to the enriched pathways in the transcriptome, the expression levels of genes related to apoptosis including nuclear factor kappa B (*nfkb*), interleukin 1 beta (*il1β*), and caspase3 (*casp3*) were measured. The data showed that the addition of xylanase decreased the gene expression of *nfkb* significantly (*P* < 0.05, Fig. 4A). Xylanase supplementation downregulated the gene expression of *il1β* (*P* < 0.05, Fig. 4B). The expression level of *casp3* which is the primary executioner of apoptotic death, was decreased significantly in SMC group compared to SM group (*P* < 0.05, Fig. 4C). The protein levels of P65 (a phosphorylated form of NF-kB) and IL-1β were also detected. The data showed that the addition of xylanase attenuated the inflammation parameters (Fig. 4D and E), suggesting that supplementation of xylanase alleviated the inflammatory response induced by soybean meal.

### Addition of xylanase altered the gut microbiota composition

**Fig. 5:**
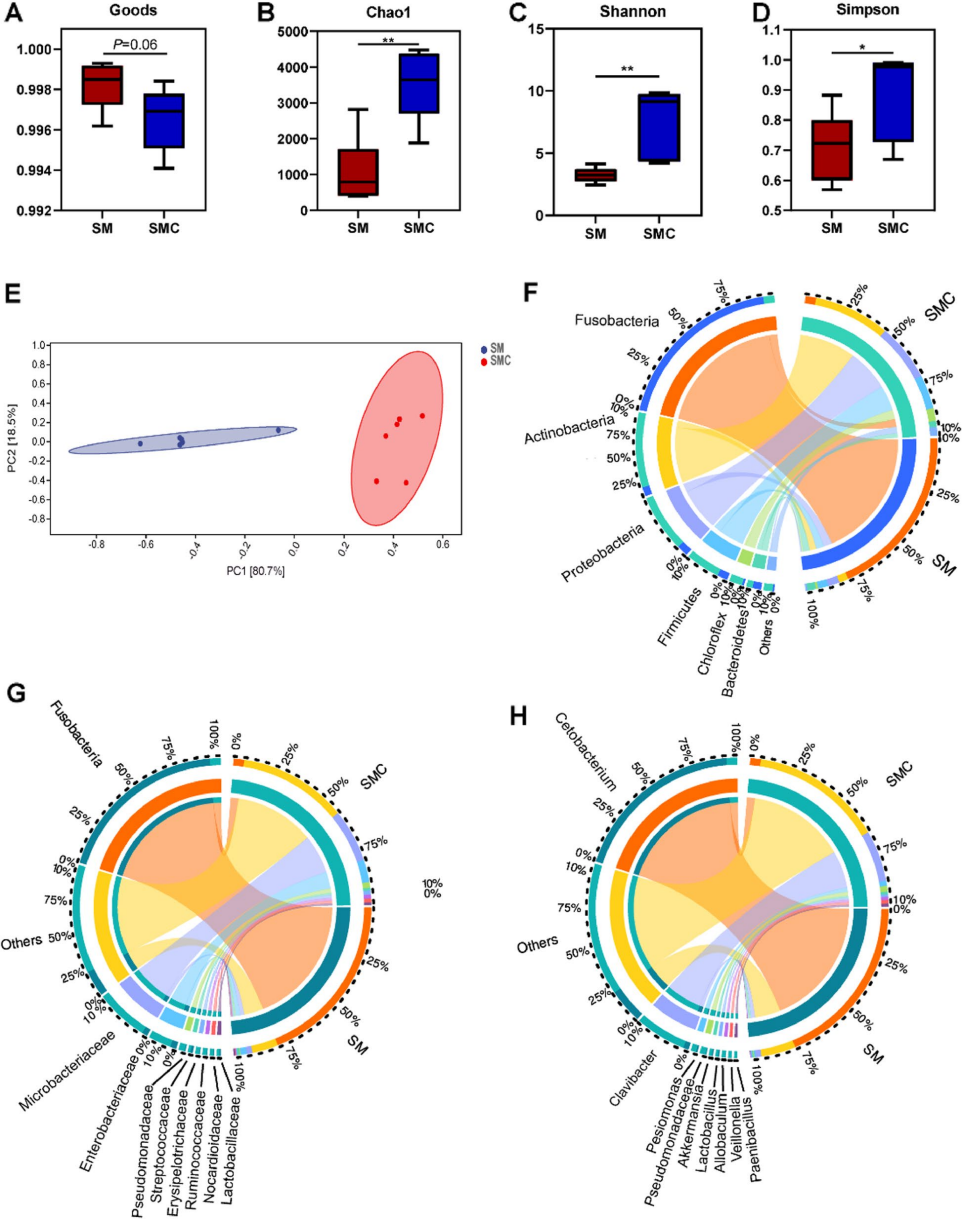
Effects of dietary supplementation with xylanase on gut microbiota composition of Nile tilapia. **A** Goods, **B** Chao1, **C** Shannon, **D** Simpson, **E** Principal component analysis (PCA), **F**–**H** Microbiota composition in phylum level, family level and genus level of each sample, respectively. Data was expressed as mean ± SEM (*n* = 6). SM, fish fed with soybean diet; SMC, fish fed with soybean diet supplemented with 3,000 U/kg xylanase. The significant differences between two group were presented at *P* < 0.05 (*), *P* < 0.01 (**)

Considering that dietary xylanase may alter intestinal microbiota composition to exert beneficial effects on host health [44, 45], high-throughput sequencing was used to investigate the effects of xylanase on gut microbiota composition in fish. Compared with SM treatment, dietary xylanase had no effects on the indices of Goods (Fig. 5A). Xylanase administration increased significantly the richness indices of Chao1 and the diversity indices of Shannon and Simpson (*P* < 0.05, Fig. 5B–D), suggesting that supplementation of xylanase in soybean meal diet improved the alpha diversity of gut microbial community. Principal component analysis (PCA) showed that bacterial communities were obviously different between SM and SMC group (Fig. 5E). In order to assess the microbial community composition of two experimental groups, we analyzed the gut microbiota composition at the phylum, family, and genus level. In SM group, the dominant phylum was Fusobacteria, while Actinobacteria, Proteobacteria and Firmicutes were the dominant phyla in SMC group (Fig. 5F). At the family level, Fusobacteriaceae was lower in SMC than in SM treatment, while Microbacteriaceae and Enterobacteriaceae were higher in SMC group (Fig. 5G). At the genus level, xylanase supplementation decreased the relative abundance of *Cetobacterium* and increased the relative abundance of *Clavibacter*, *Akkermansia,* and *Lactobacillus* compared to soybean meal diet (Fig. 5H). Collectively, dietary xylanase altered the gut microbiota composition and increased intestinal microbial diversity.

### Xylanase increased the content of butyric acid in the gut

**Fig. 6:**
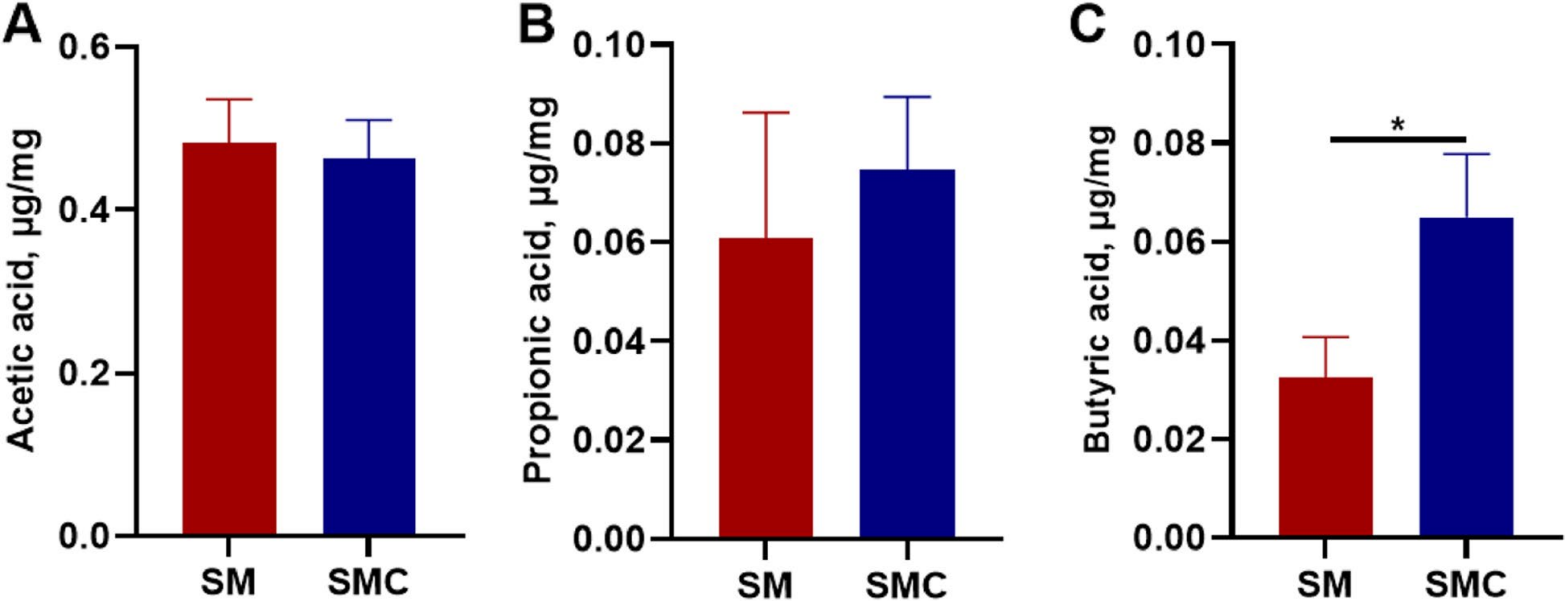
Effects of dietary supplementation with xylanase on SCFAs in gut of Nile tilapia. **A** Acetic acid, **B** Propionic acid, **C** Butyric acid. Data was expressed as mean ± SEM (*n* = 6). SM, fish fed with soybean diet; SMC, fish fed with soybean diet supplemented with 3,000 U/kg xylanase. The significant differences between two group were presented at *P* < 0.05 (*)

The gut microbiota can utilize the nutrients to produce beneficial metabolites such as short-chain fatty acids (SCFAs). Considering the change in gut microbiota composition in SMC group, we measured the production of SCFAs in the gut. Compared with SM treatment, xylanase supplementation did not influence the content of acetic acid or propionic acid (Fig. 6A and B), but it increased significantly the production of butyric acid (*P* < 0.05, Fig. 6C). The above data indicated that addition of xylanase in soybean meal increased the concentration of butyric acid in the gut of Nile tilapia.

### Sodium butyrate mirrored the effects of xylanase in Nile tilapia

**Fig. 7:**
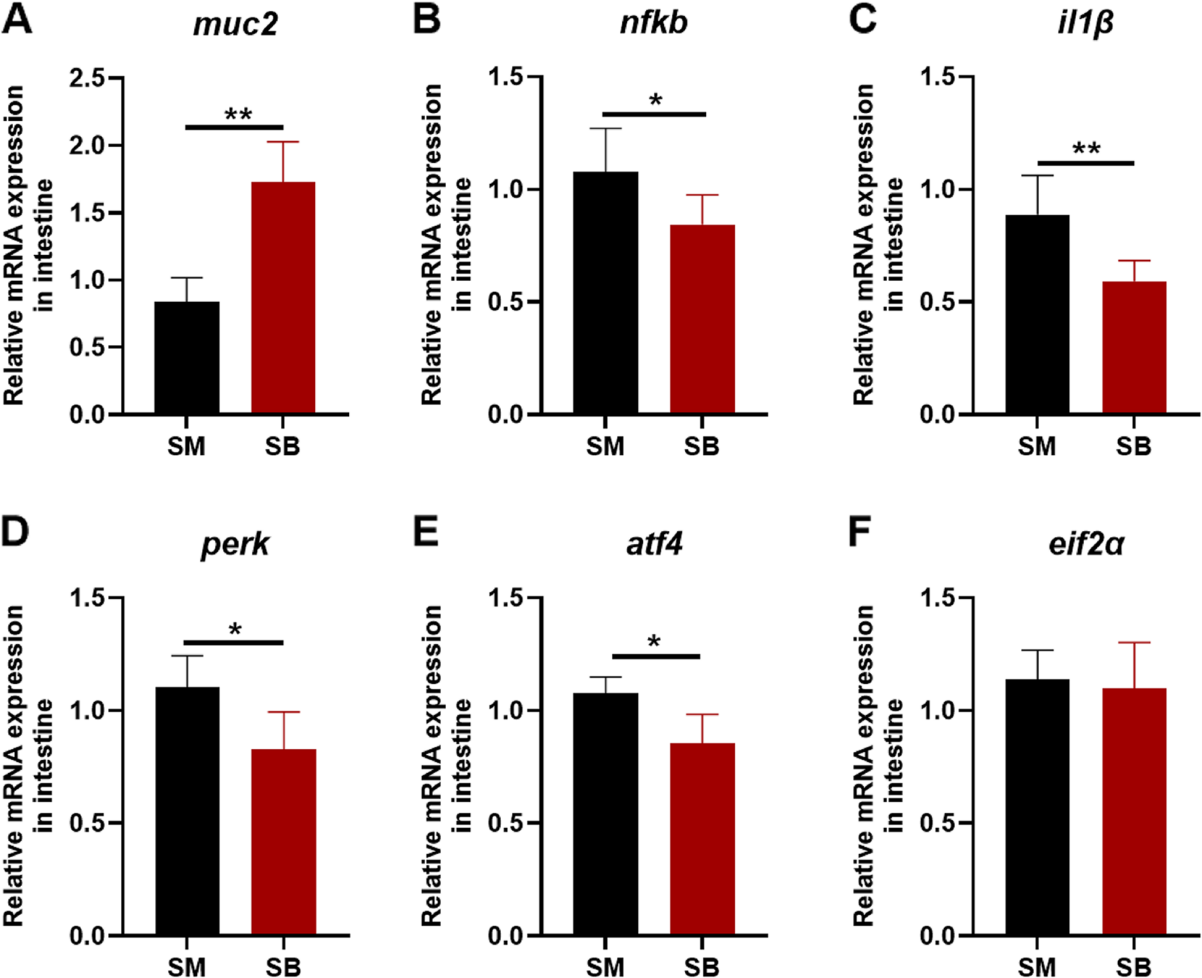
Effects of dietary sodium butyrate on endoplasmic reticulum (ER) stress and inflammation response in Nile tilapia. Gene level of **A** Mucin2 (*muc2*), **B** Nuclear factor kappa B (*nfkb*), **C** Interleukin 1 beta (*il1β*), **D** Protein kinase RNA-like endoplasmic reticulum kinase (*perk*), **E** Activating transcription factor 4 (*atf4*), **F** Initiation factor 2 alpha (*eif2α*). Data was expressed as mean ± SEM (*n* = 6). SM, fish fed with soybean diet; SB, fish fed with soybean diet supplemented with 40 mmol/kg sodium butyrate. The significant differences between two group were presented at *P* < 0.05 (*), *P* < 0.01 (**)

To verify whether butyric acid can mirror the effects of xylanase, Nile tilapia were fed with a soybean meal diet with or without 40 mmol/kg sodium butyrate (SB) for 6 weeks. The addition of sodium butyrate in soybean meal upregulated the gene expression of *muc2* significantly (*P* < 0.05, Fig. 7A). Dietary sodium butyrate obviously reduced the expression level of *nfkb* and *il1β* compared with SM treatment (*P* < 0.05, Fig. 7B and C), and decreased the expression levels of *perk* and *atf4* in Nile tilapia (*P* < 0.05, Fig. 7D and E). In addition, sodium butyrate did not influence the expression level of *eif2α* (Fig. 7F). Collectively, dietary sodium butyrate in soybean meal increased the expression of *muc2* and inhibited ER stress or inflammation of Nile tilapia.

## Discussion

The NSP in soybean meal cannot be digested by the endogenous enzymes, so the remaining NSP in the diet increased the digesta viscosity which caused negative effects on the growth performance of animals [46]. Xylanase hydrolyzes xylan to xylooligosaccharides or xylose, but the overproduction of xylooligosaccharides or xylose may also decrease the weight gain in high level xylanase treatment [31]. It has been found that addition of xylanase in the plant meal diet at 594–1,880 U/kg increased the final body weight, but the xylanase higher than 1,880 U/kg would decrease the body weight of Jian carp gradually [34]. The concentration dependent effect of xylanase in promoting growth was also found in juvenile mori (*Cirrhinus mrigala*) fed with plant-based diets [25]. It has been found that supplementation of dietary xylanase at 4,228 U/kg and 1,120 TXU/kg in the diet increased the final body weight of Nile tilapia [47, 48]. In the present study, we found addition of 1,500 U/ kg xylanase in soybean meal did not affect the final weight (data not shown), while 3,000 U/kg dietary xylanase administration in soybean meal decreased the weight gain of Nile tilapia. The decrease in weight gain was mainly caused by the decreased viscerosomatic, but the weight of carcass was increased in xylanase treatment, suggesting that xylanase increased the proportion of edible parts in Nile tilapia. It has been reported that carcass yield was improved by xylanase administration in broiler [49, 50]. The above data suggested that except for the enzyme concentration, the body condition parameters should also be considered when we fully evaluate the influence of xylanase on the growth performance of fish [51].

The epithelium and intestinal mucosa act as physical barriers for the host's defense against pathogen infection, and an intact intestinal barrier is critical for maintaining host health [52]. High levels of soybean meal containing NSP had negative impacts on the gut barrier, including impaired villi morphology, decreased number of goblet cells, and increased intestinal permeability [53]. Previous study found that supplementation of xylanase in sunflower meal enhanced the height of mucosal folds, and mucosa thickness of the intestine in Nile tilapia [27]. Adeoye et al. reported that dietary exogenous xylanase enhanced gut health by increasing the microvilli density of Nile tilapia [54], which is consistent with our results. However, the mechanism by which xylanase improves gut health remains unclear.

Supplementation of xylanase can degrade NSP in diet to produce oligosaccharides. The released oligosaccharides might be fermented by gut microbiota to promote bacterial proliferation. In the research of large yellow croaker, xylanase administration increased the richness of gut microbiota but had no effect on the diversity of gut microbiota [28]. For African catfish (*Clarias gariepinus*), supplementation of dietary xylanase in plant-based diets changed the hindgut microbial diversity [55]. In the present work, xylanase increased both richness and diversity of gut microbiota in Nile tilapia. Dietary xylanase decreased the abundance of Fusobacteria, but increased the abundance of Actinobacteria, Proteobacteria, and Firmicutes. In line with this result, Jiang et al*.* also reported that xylanase enhanced the abundance of *Lactobacilli* and depressed the abundance of *E. coli* and *Aeromonas* [31]. The above researches suggested that xylanase can change the intestinal microbiota composition.

Intestinal microbiota can utilize the oligosaccharides to produce SCFAs, so the changes in the intestinal microbiota may affect the content of intestinal metabolites. Dietary xylanase supplemented in a vegetable-based diet increased the abundance of *Bifidobacterium* which produced more acetate and butyrate in Nile tilapia [56]. In the present study, xylanase altered the composition of gut microbiota in Nile tilapia and increased the abundance of *Akkermansia* which can produce butyrate [57, 58]. Further analysis confirmed that the addition of xylanase in soybean meal increased the content of butyrate, which is the energy source for fish gut [59]. Thus, the increased butyrate concentration may account for the improved gut morphology and function of Nile tilapia in xylanase supplemented group.

Dietary butyrate can improve gut health and upregulate the expression of genes related to tight junction proteins to enhance gut barrier function in grass carp, marine teleost (*Sparus aurata*), turbot (*Scophthalmus maximus* L.), rainbow trout (*Oncorhynchus mykiss*) [60–63]. In the present study, dietary xylanase supplementation upregulated the expression of MUC2 which codes for the typical mucin. The expression of MUC2 is regulated by various factors, including gut microbiota and SCFAs [64–66]. It has been reported the addition of butyrate in plant-food-based diets improved the gut health of fish by increasing the number of goblet cells which can secret more mucins [67–69]. The expression level of *muc2* had an increased tendency fed with plant- based diets containing sodium butyrate in European sea bass (*Dicentrarchus Labrax*) [70]. In mammals, supplementation of butyrate stimulated the production of MUC2 to improve intestinal health [71, 72]. In the present study, we also found that dietary butyrate supplemented in a soybean meal diet upregulated the expression of *muc2*, suggesting the function of butyrate in increasing *muc2* expression is similar in different hosts.

MUC2 is N-glycosylated in the ER. The disruption of ER function by exogenous stimuli leads to the accumulation of unfolded or misfolded proteins and then reduces the secretion of MUC2 [73]. High concentration of soybean protein can increase the expression of *perk, eif2α,* and *atf4,* suggesting the existence of endoplasmic reticulum stress in the intestine of pacific white shrimp (*Litopenaeus vannamei*) [74]. In the present study, we found that supplementation of xylanase inhibited *perk*/*atf4* signaling pathway to alleviate ER stress induced by soybean meal, which may be related to the increased expression of MUC2. Although the interaction between xylanase and ER function remains unknown in aquaculture, it has been indicated that butyrate can inhibit the *ire1α/xbp1* pathway to reduce soybean meal-induced ER stress in the intestine injury [75]. Our results also revealed that the addition of butyrate in soybean meal can repress the *perk*/*atf4* signaling pathway to relieve the ER stress which may account for the increase of *muc2* expression.

## Conclusion

In conclusion, the present study indicated that supplementation of xylanase in soybean meal altered the composition of gut microbiota and increased the concentration of butyrate which increased the expression of *muc2* to enhance the gut barrier function in Nile tilapia. The present study provides important mechanistic insights into the interactions between xylanase and the gut barrier in fish and the study indicated that xylanase can act as feed additives to alleviate the adverse effects of NSP. In addition, this work also provides a theoretical basis for the application of xylanase in plant protein feed.

## Abbreviations

ANFs: Antinutritional factors
ASV: Amplicon sequence variants
atf4: Activating transcription factor 4
casp3: Caspase3
CHOP: C/EBP homologous protein
ef1α: Elongation factor 1 alpha
eif2α: Eukaryotic initiation factor 2 alpha
ELISA: Enzyme-linked immunosorbent assay
GRP78: Glucose regulatory protein 78
H&E: Hematoxylin and eosin
il1β: Interleukin 1 beta
LPS: Lipopolysaccharide
MUC2: Mucin2
nfkb: Nuclear factor kappa B
NSP: Non-starch polysaccharides
PCA: Principal component analysis
perk: Protein kinase RNA-like endoplasmic reticulum kinase
qRT-PCR: Quantitative real-time polymerase chain reaction
SB: Sodium butyrate
SCFAs: Short chain fatty acids
SEM: Standard error of the mean
SM: Soybean meal
SMC: Soybean meal + 3,000U/kg xylanase
XOS: Xylooligosaccharides
